# Evaluation of ELISA for the detection of rabies virus antibodies from the thoracic liquid and muscle extract samples in the monitoring of fox oral vaccination campaigns

**DOI:** 10.1186/s12917-016-0701-0

**Published:** 2016-05-10

**Authors:** Tomislav Bedeković, Ivana Šimić, Nina Krešić, Ivana Lojkić, Željko Mihaljević, Ivica Sučec, Ivana Lohman Janković, Peter Hostnik

**Affiliations:** Croatian Veterinary Institute, Savska cesta 143, 10000 Zagreb, Croatia; University of Ljubljana, Veterinary Faculty, Gerbičeva 60, 1115 Ljubljana, Slovenia; Veterinary Directorate, Planinska 2a, 10000 Zagreb, Croatia

**Keywords:** BioPro ELISA, Muscle extract, Thoracic liquid, Evaluation

## Abstract

**Background:**

The main goal of oral vaccination of foxes is eradication of rabies in the red fox population as rabies reservoirs. To evaluate the success of vaccination a serological testing is conducted as a part of monitoring program. Two different methods are used regarding rabies serology: virus neutralisation test and ELISA.

**Methods:**

In this study the reliability of BioPro ELISA was evaluated for testing haemolytic thoracic liquids and muscle extracts originated from 147 foxes in comparison to mFAVN. Also, the influence of heat treatment of samples on test results was investigated.

**Results:**

The specificity of the test for not-heat treated samples was 92.98 % and sensitivity 79.20 %. Diagnostic validity of the ELISA compared to the mFAVN test when not-heat treated samples were used was 89.16 %. The specificity of the test for heat treated samples was 79.10 % and sensitivity 96.36 %. Diagnostic validity of the BioPro ELISA compared to the mFAVN test for heat treated samples was 94.30 %.

**Conclusion:**

According to this study, the BioPro ELISA is reliable tool for detection of rabies specific antibodies in the context of evaluation of oral vaccination of foxes from poor quality samples as a substitution for virus neutralisation tests.

## Background

In Europe, the main goal of oral vaccination (ORV) is eradication of rabies in wildlife [1]. To achieve this objective, oral vaccine baits (vaccine Lysvulpen, Bioveta, Czech Republic) containing live attenuated rabies virus is used in Croatia. The ORV of foxes in Croatia was carried out simultaneously with similar programs in the neighbouring countries as a part of control and eradication of rabies in Western Balkan countries co-financed by European Commission [2]. As a part of monitoring, vaccination efficacy is evaluated considering the rabies incidence, bait uptake and humoral immune response [1]. This approach showed very good results in effectiveness of elimination of terrestrial rabies in Europe [3].

Considering the assessment of vaccination efficiency the main obstacle in evaluation of humoral response is the low quality of the samples. The quality of the samples depends on the sampling procedure. In some countries the sampling is organised immediately after killing of foxes, but in most countries the carcasses of foxes are frozen before sampling. Time between shooting and sampling in the lab can be extended (up to 10 days). In such case it is difficult to collect serum from the foxes. Consequently, it is not possible to perform serological test and evaluate humoral immunological response. As a substitution for the serum, the liquid from thoracic cavity or extracts of the muscles can be used as samples [4].

Currently two different methods are used regarding rabies serology: virus neutralisation test and ELISA. Neutralisation tests: fluorescent antibody virus neutralisation (FAVN) test and rapid fluorescent focus inhibition test (RFFIT) are known to be the most reliable tests for evaluation of successful vaccination [5–8]. However, the neutralisation tests are time consuming, expensive and require live rabies virus [9]. Also, sometimes the results cannot be read off due to cytotoxic effect on the cells [10]. In order to overcome that problem the ELISA has been developed. Currently two ELISAs for detection of rabies specific antibodies existed on the European Union market: Bio-Rad (Platelia Rabies II - Biorad, Marnes-La-Coquette, France) and BioPro (Prague, Czech Republic). BioPro ELISA is recently developed test especially designed for the detection of rabies virus antibodies in the monitoring of the fox ORV campaigns [9]. This test was evaluated on fresh foxes samples and also on dogs and cats samples [9, 11] and showed to be a reliable tool for serological testing. Until now, muscle extracts and poor quality thoracic liquid samples were not used as samples for serological testing with virus neutralisation tests. The main reason for that was cytotoxic effect caused by quality of the samples. Because of the same reason it was also impossible to evaluate ELISA on those samples. Recently, the new test – modified fluorescent virus neutralisation test (mFAVN) was developed and problem with cytotoxicity has been eliminated [4]. Furthermore, according to that study, very good agreement between antibody titre in dog sera, thoracic liquids and muscle extracts were recorded. Those findings allow evaluation of ELISA for the detection of rabies virus antibodies from the thoracic liquid and muscle extract samples in the monitoring of fox ORV campaigns.

This paper evaluates the specificity and sensitivity of commercial BioPro ELISA kit for the detection of rabies virus antibodies from the haemolytic thoracic liquid and muscle extract samples in the monitoring of fox ORV campaigns. Furthermore, the possibility to improve diagnostic validity of the ELISA by heat treatment of the samples was investigated.

## Methods

### Ethics statement

All samples were collected by hunters in compliance with national regulations according to “Order on oral vaccination of foxes on territory of Republic of Croatia” [12]. Sampling was performed strictly as a part of program “Control and eradication of rabies in Croatia” co-financed by IPA project (number of project: 2008-0303-0804). Sampling was approved by ethics committee of Croatian Veterinary Institute (decision number: Z-VI-4-2261-1/12).

### Samples

The samples were collected from the 147 foxes during the monitoring of fourth ORV campaign (autumn 2012). The first choice for sampling was thoracic liquid. However, in the case that thoracic liquid could not be collected the piece of *m. femoralis* was taken in order to obtain muscle extract. So, the muscle and thoracic liquid samples were never taken from the same animal. Therefore, from the 55 foxes the fluid from the thoracic cavity was taken and from 92 foxes the piece of *m. femoralis* (approximately 5 × 7 cm) was taken. The carcasses of foxes were not frozen before sampling. The carcasses were placed at ambient temperature and sampled 3–4 days post mortem.

The collected haemolytic liquid samples from the thoracic cavity were centrifuged at 220 × g for 10 min and the separated liquid was placed in two sterile tubes and stored at −20 °C until testing. In order to collect muscle extracts, the muscles in sterile flasks were frozen at - 20 °C for 4 days and then placed at 4 °C for 3–5 days. From each piece of muscle, a sample approximately 200–300 μl of the muscle extract was collected, centrifuged at 220 × g for 10 min, placed in two sterile tubes and stored at −20 °C prior to analysis. On the day of testing, from each sample one tube was heat-treated at 56 °C for 30 min and centrifuged (220 × g). Before testing, all samples were centrifuged one more time.

The heat treated samples were tested using the both tests: mFAVN test and BioPro ELISA. The not-heat treated samples were tested only using the BioPro ELISA. The not-heat treated samples were not tested with mFAVN test because in that case unspecific reactions could be observed [13].

### Virus neutralisation test

Rabies neutralising antibodies were detected in thoracic liquids and muscle extracts with the mFAVN as described previously [4]. In that paper mFAVN test was evaluated using the dog samples with the cut off 0.5 IU/ml. However, in this paper instead of the term “positive” (0.5 IU/ml) the term “threshold of detection” (0.1 IU/ml) in the context of evaluation of fox ORV was adopted. Because of that, for the purpose of this study the mFAVN test was compared with FAVN test on the dog samples as described previously [4] and evaluated using the cut of 0.1 IU/ml.

### ELISA

The rabies antibodies were detected in thoracic liquids and muscles extracts with the BioPro Rabies ELISA kit according to the manufacturer instructions. The BioPro ELISA is blocking ELISA for detection of rabies virus antibodies in serum or plasma. The wells of microplates are coated with rabies antigen. Diluted samples are incubated in the wells. After washing biotinylated anti-rabies antibody is added to wells. In the case of positive samples specific antibodies will block binding of biotynylated anti-rabies antibodies with coated rabies antigen. The conditions of validation described by the manufacturer were implemented to interpret the results obtained for the samples. The percentage of blocking was calculated for each sample according to the manufacturer’s specifications. For checking the effectiveness of ORV campaigns, the manufacturer was established the threshold of positivity to 40 %.

### Statistical analysis

The true positive and true negative test results were determined by the mFAVN. Evaluation of ELISA and results were interpreted according to previous studies [14, 15]. The validity or degree to which the test measures what it claims to measure is assessed by area under ROC curve using STATA 10 (Stata Press, College station, Texas, USA). The agreement beyond chance level between the two diagnostic tests (kappa value), and measurements of the test performance area under the ROC curve were calculated using STATA 10 (Stata Press, College station, Texas, USA).

## Results

### Virus neutralisation test

In this paper complete neutralization at serum dilution above of 1:4 was corresponding to titer 0.1 IU/ml. The validation of mFAVN test for difference cut-off was performed using the results from the previous study [4]. The sensitivity and specificity of the mFAVN test compared to FAVN test for difference cut-off are recorded in Table 1. Diagnostic agreement (when cut-off was set on 0.1 IU/ml in both test) between FAVN and mFAVN tests of serum was perfect.

**Table 1:** The sensitivity and specificity for the mFAVN test compared to FAVN test for different cut-offs

	Cut-off value	Sensitivity	Specificity	Correctly classified	Cumulative AUC
1.	0.100	1.0000	1.0000		
2.	0.200	1.0000	1.0000		
3.	0.300	1.0000	0.9655	99.0000	0.9976
4.	0.400	0.9706	0.8750	94.0000	0.9910
5.	0.500	0.9440	0.8880	94.0000	0.9900

### BioPro ELISA

To establish the specificity and sensitivity of the ELISA, 147 samples (thoracic liquids and muscle extracts) were tested in parallel with the mFAVN and ELISA (Table 2). No cytotoxicity was observed for either type of samples when mFAVN were used.

**Table 2:** Results of testing for muscle extracts and thoracic liquids

	mFAVN test		ELISA		ELISA	
			Not-heat-treated		Heat-treated	
	Positive	Negative	Positive	Negative	Positive	Negative
Thoracic liquid	38	17	36	19	40	15
Muscle extract	15	77	5	87	23	69
Total	53	94	41	106	63	84

The cut –off for mFAVN test was set on 0.1 IU/ml

The cut –off for BioPro ELISA was set on 40 %

**Fig. 1:**
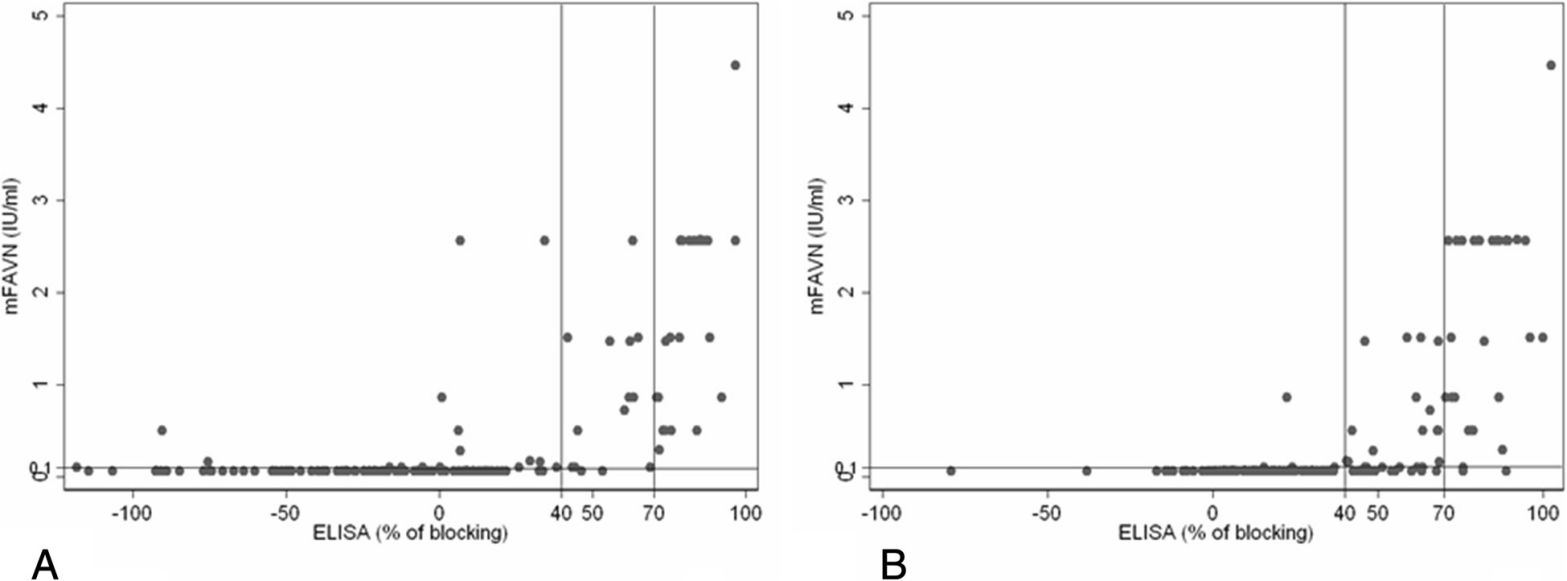
**a** Correlation between titres of antibodies (IU/ml) in not-heat treated samples titrated by mFAVN test (cut off was set on 0.1 IU/ml) and percent of blocking obtained with BioPro ELISA (cut off was set on 40 %). **b** Correlation between titres of antibodies (IU/ml) in heat treated samples titrated by mFAVN test (cut off was set on 0.1 IU/ml) and percent of blocking obtained with BioPro ELISA (cut off was set on 40 %)

**Fig. 2:**
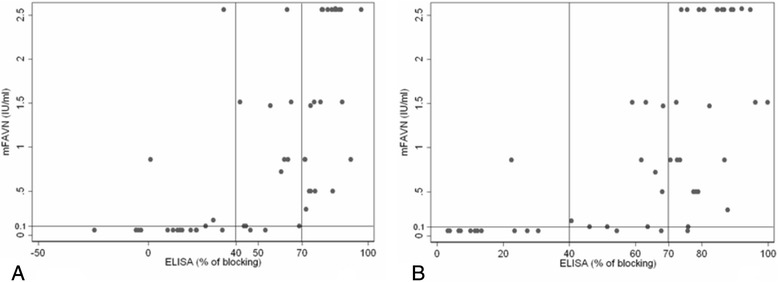
**a** Correlation between titres of antibodies (IU/ml) in not-heat treated thoracic liquids samples titrated by mFAVN test (cut off was set on 0.1 IU/ml) and percent of blocking obtained with BioPro ELISA (cut off was set on 40 %). **b** Correlation between titres of antibodies (IU/ml) in heat treated thoracic liquids samples titrated by mFAVN test (cut off was set on 0.1 IU/ml) and percent of blocking obtained with BioPro ELISA (cut off was set on 40 %)

**Fig. 3:**
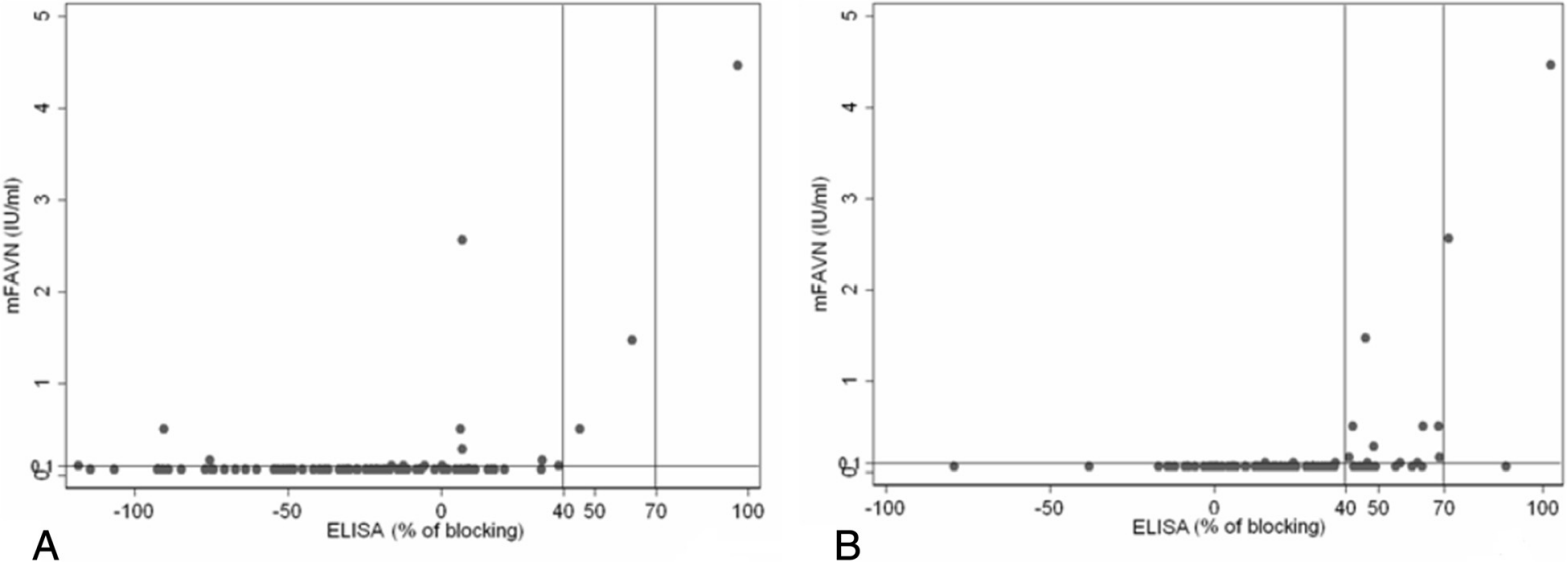
**a** Correlation between titres of antibodies (IU/ml) in not-heat treated muscle extracts samples titrated by mFAVN test (cut off was set on 0.1 IU/ml) and percent of blocking obtained with BioPro ELISA (cut off was set on 40 %). **b** Correlation between titres of antibodies (IU/ml) in heat treated muscle extracts samples titrated by mFAVN (cut off was set on 0.1 IU/ml) and percent of blocking obtained with BioPro ELISA (cut off was set on 40 %)

The results regarding sensitivity, specificity, diagnostic agreement and diagnostic validity for either type of samples are recorded in Table 3. Correlation between titres of antibodies titrated by mFAVN test and percent of blocking obtained with BioPro ELISA are recorded in Figs. 1, 2 and 3.

**Table 3:** Results of sensitivity, specificity, diagnostic agreement and diagnostic validity for BioPro ELISA

	All samples		Muscle extracts		Thoracic liquids	
	Not-heat treated treated	Heat	Not-heat treated treated	Heat	Not-heat treated treated	Heat
Specificity	92.98 %	79.10 %	100 %	87.50 %	89.47 %	85.00 %
Sensitivity	79.20 %	96.36 %	60 %	83.33 %	90.00 %	97.43 %
Diagnostic agreement	89.12 %	87.76 %	89.13 %	84.78 %	89.09 %	92.73 %
Diagnostic validity	89.16 %	94.3 %	70.30 %	88.70 %	95.98 %	94.90 %
Confidential interval	0.825–0.958	0.906–0.979	0.519–0.886	0.801–0.973	0.912–1.00	0.888–1.00
Kappa *p* < 0.001	0.7517	0.7449	0.4560	0.5410	0.7526	0.8240

## Discussion

The most effective method for evaluation of ORV campaign success is monitoring program. [1]. Main parts of program are control of bait uptake (tetracycline detection) and evaluation of immunological response (detection of specific antibodies) [16]. The specific rabies antibodies in the monitoring of fox ORV campaigns can be detected by using two different methods: virus neutralisation test and ELISA [16]. ELISA is simpler and faster method but the virus neutralization test is still considered as a golden standard. Although the principle of ELISA is different compared to virus neutralization test for the validation purpose the ELISA is usually compared with the virus neutralisation test. However, the possibility of evaluation depends on quality of samples. The poor quality samples may prevent the successful performance of virus neutralisation test. Because of that ELISA has never been evaluated for detection of antibodies against rabies in muscle extracts. Recently, the ELISA was evaluated on fresh collected thoracic liquid samples of foxes [11] and on sera samples of dogs and cats [9].

In this study ELISA was evaluated in the part of ORV campaigns monitoring as a tool for detection of specific rabies antibodies in poor quality hemolytic thoracic liquids and muscle extract samples. For that purpose the term “threshold of detection” (0.1 IU/ml) was applied instead of generally accepted adequate immunological response after vaccination (0.5 IU/ml). As is mentioned previously [17], because no specific rabies virus neutralisation antibodies level can been identified as absolute protection under all circumstances and in all hosts against all rabies virus variant infections, antibodies level attained by the majority of subjects in vaccine clinical trials formed the basis for the levels currently recognized as the minimal adequate response in vaccinated humans. According to World Health Organisation (WHO) that minimal adequate response is 0.5 [18]. This level was also adopted from World Organisation for Animal Health (OIE) as a minimal adequate response in vaccinated animals [13]. However, according to United States Advisory Committee on Immunization Practices (US ACIP) the adequate response is if no nonspecific inhibition reactions were found in serum dilutions above 1:5 [19]. The level described in the US ACIP is approximately 0.1 IU/ml in the RFFIT as originally described [20]. Furthermore, the main point regarding ORV serological monitoring is that for the purpose of detection of immunological response, a lower level is better suited than the globally recognized 0.5 IU/ml level for proof of adequate response to vaccination. Also, according to the previous study [17] the titer 0.1 IU/ml corresponding to a complete neutralisation at 1:5 serum dilution in the RFFIT. However, according to the same authors this can represent different titers and IU/ml values in laboratories that perform modified RFFIT which is the case with the mFAVN test described in this paper. In addition, United States Department of Agriculture considers an even lower cut-off >50 % neutralization at the 1:5 serum dilution when evaluating oral vaccination (~0.05 IU/ml) [21]. Also, according to manufacturer’s instructions in the context of evaluation of fox ORV results the threshold of positivity of ELISA is decreased from the 70 to 40 %. According to manufacturer’s instructions the percentage of blocking of 70 % is equivalent to 0.5 IU/ml obtained with virus neutralisation test. According to that, the percent of blocking 40 % in ELISA is lower than 0.5 IU/ml obtained with virus neutralisation test. In this study the purpose of detection of rabies virus antibodies in the monitoring of fox ORV campaigns was to detect immunological response on vaccination but not to consider the WHO or OIE minimal adequate response (0.5 IU/ml) [11]. Also, according to previous study the level of antibodies in muscle extracts and haemolytic thoracic liquids was lower compared to serum samples [4]. Because of the all above mentioned facts, the threshold of detection of virus neutralisation test as a part of monitoring of ORV campaigns was decreased to 0.1 IU/ml in order to establish the adequate immunological response to vaccination which can be the proof of seroconversion [20]. However, before the testing the mFAVN test was validated in comparison to FAVN test using difference cut-offs. As are recorded in Table 1 the sensitivity and specificity of the mFAVN compared with FAVN test were even higher for cut-off 0.1 IU/ml compared to cut off 0.5 IU/ml and show perfect agreement.

According to results in this study, the agreement between BioPro ELISA and mFAVN was 89.12 % for not-heat treated samples, and 87.76 % for heat treated samples. This means that heat treatment of the samples has not influence on kappa value of the BioPro ELISA test. Also, heat treatment of the samples just slightly increases a diagnostic validity of the test. However, if we compare an agreement of BioPro ELISA and mFAVN for thoracic liquids and muscle extracts separately, results are different.

According to previous study performed on foxes [11] the agreement between the BioPro ELISA and virus neutralisation test was 95.10 %. In that study thoracic liquid from the carcasses of the fresh foxes were used as a sample. In our study samples were not collect from the fresh foxes and because of that extremely haemolytic thoracic liquid samples were used for testing. The agreement between the BioPro ELISA and the mFAVN test was 89.09 % for not-heat treated and 92.73 % for heat treated samples. So, according to results in our study the quality of thoracic liquid samples does not have influence on results obtained with BioPro ELISA or it insignificantly decreases the agreement between tests. Also, when thoracic liquids were used, heat treatment does not increase the validity of the test.

The opposite situation was recorded when muscle extracts were used as a sample. Even though diagnostic agreement between ELISA and mFAVN for heat treated samples is slightly decreased (4 %) the validity of the test is significantly increased (18 %). The reason for that is that according to this study heat treatment of the muscle extracts causes increase in sensitivity of the test but also causes decrease in test specificity. This finding is expected because in general, the higher the sensitivity, the lower the specificity, and vice versa. However, in this study the improvement in sensitivity was significantly higher compared to specificity decreasing which result in increase of the test validity. The lower sensitivity when not-heat treated muscle extracts were used as samples is very important; in that case a lot of false negative results could be obtained with ELISA. The reason for different influence of heat treatment on thoracic liquids and muscle extracts can be explained in different chemical composition of muscle (enzymes, proteins) compared to blood. Also, the reason for decreasing of the test specificity can be due to influence of heat treatment on specific antibody structure (proteins) or antibody activity. However, the influence of heat treatment on test specificity and sensitivity should be further investigated.

According to this study the kappa value ranged between 0.4560 for not-heat treated muscle extract samples to 0.8240 for thoracic liquids heat treated samples. Overall, the kappa value was significantly lower for muscle extracts compared to thoracic liquids and indicated only moderate agreement of the test when muscle extracts were used as samples. However, the more relevant measure is the test validity or degree to which the test measures what it claims to measure. The kappa value only considers the cut offs but the diagnostic validity considers all results and provides better overview of test. According to our study the diagnostic validity for all samples except not-heat treated muscle extracts was very high (88.70–95.98 %) and indicated very good reliability of the BioPro ELISA test.

## Conclusions

According to this study haemolytic thoracic liquids and muscle extracts can be used as samples for detection of specific rabies antibodies with BioPro ELISA. Muscle extracts samples should be heat treated before testing in order to increase the validity of the test.

## Ethics and consent to participate

This study was performed in compliance with national guidelines according to “Order on oral vaccination of foxes on territory of Republic of Croatia” – Official gazette, number 47, 2012, Croatia, available at: http://narodne-novine.nn.hr/.

Sampling was approved by ethics committee of Croatian Veterinary Institute (decision number: Z-VI-4-2261-1/12).

### Consent to publish

Not applicable.

## Availability of data and materials

All row data are available in Laboratory for rabies at Croatian veterinary Institute upon official request via mail (bedekovic@veinst.hr).

## Abbreviations

ACIP: Advisory Committee on Immunization Practices
ELISA: enzyme linked immunosorbent assay
FAVN test: fluorescent antibody virus neutralisation test
IU/ml: international unit/millilitre
mFAVN test: modified fluorescent antibody virus neutralisation test
RFFIT: rapid fluorescent focus inhibition test
WHO: World Health Organisation
